# Expression of programmed cell death ligand 1 protein and other biomarkers in patients with gastric cancer and gastroesophageal junction cancer: a retrospective single centre study in Brazil

**DOI:** 10.3389/fonc.2025.1623264

**Published:** 2025-09-10

**Authors:** Jéssica Gonçalves Azevedo, Beatriz de Araujo Cortez, Maria Aparecida do Carmo Rego, Felipe Berlinski, Dominihemberg Ferreira, Angélica Carreira dos Santos, Ana Beatriz Machado De Almeida, Paula de Mendonça Batista, Cicera Pimenta Marcelino, Fernanda Franco Munari, Iara Viana Vidigal Santana, Vinicius Duval da Silva, Guilherme Ribeiro, Gustavo Noriz Berardinelli, Diego Burgardt, Durval R. Wohnrath, Rui Manuel Reis

**Affiliations:** ^1^ Global Medical & Scientific Affairs (GMSA), MSD Brazil, São Paulo, Brazil; ^2^ RWE Brazil, IQVIA, São Paulo, Brazil; ^3^ Molecular Oncology Research Center, Barretos Cancer Hospital, São Paulo, Brazil; ^4^ Department of Pathology, Barretos Cancer Hospital, Barretos, São Paulo, Brazil; ^5^ Molecular Diagnostic Laboratory, Barretos Cancer Hospital, Barretos, São Paulo, Brazil; ^6^ Department of Upper Digestive Surgery, Barretos Cancer Hospital, São Paulo, Brazil; ^7^ Life and Health Sciences Research Institute (ICVS), School of Medicine, University of Minho, Braga, Portugal

**Keywords:** programmed cell death ligand 1, microsatellite instability, human epidermal growth factor receptor 2, gastric cancer, gastroesophageal junction cancer, real-world data; Brazil

## Abstract

**Background:**

Programmed cell death ligand 1 (PD-L1) is a key prognostic biomarker that can predict response to immunotherapies in patients with gastric cancer (GC) and gastroesophageal junction cancer (GEJC). However, there is a lack of real-world data on the distribution of PD-L1 and other prognostic biomarkers among patients with GC and GEJC in Brazil.

**Objectives:**

To analyze PD-L1 expression, the microsatellite instability (MSI) and human epidermal growth factor receptor 2 (HER-2) status among patients with GC and GEJC in a Brazilian cancer hospital and to evaluate the association between PD-L1 expression and other biomarkers and clinicopathological parameters.

**Methods:**

This observational, retrospective study was conducted between March 2019 and May 2019 at the *Barretos Cancer Hospital* in Brazil. The levels of PD-L1 expression and other biomarkers were analyzed for patients whose formalin-fixed paraffin-embedded tumor tissue samples were preserved at the hospital. PD-L1 expression was measured by the immunohistochemical (IHC) method. MSI was determined by molecular assays, whereas IHC and fluorescence *in situ* hybridization (FISH) assays were conducted to evaluate HER-2 expression. The association between PD-L1 expression, MSI, HER-2-positivity, and clinicopathological parameters was determined using a chi-square test.

**Results:**

A total of 162 patients were included in the study. Most of the patients were male (65.4%), with a mean age of 61 years. PD-L1 expression (CPS ≥1) was observed in 49.4% of patients (n = 80) of patients, whereas MSI-high and HER-2 expression were reported in 12.3% (n = 20) and 8.0% (n = 13), respectively. PD-L1 expression was significantly associated with older age and MSI.

**Conclusion:**

A high prevalence of PD-L1 expression was observed among patients with GC and GEJC, but HER-2-positivity was lower than global prevalence. PD-L1 expression was associated with MSI-high status. The study outcomes can be used for the selection of appropriate therapies for patients with GC and GEJC in Brazil.

## 1 Introduction

Gastric cancer (GC) is the sixth most common cancer and the sixth leading cause of cancer mortality worldwide (1). In 2022, approximately 968,000 new cases and 660,000 deaths from GC were reported globally (1). The incidence and mortality rates associated with GC are highly variable among Latin American countries, ranging from 2.8 to 14.3 age-standardized rate (ASR) per 100,000 and 2.4 to 11.1 ASR per 100,000, respectively (1). In Brazil, 14,700 men and 8,321 women were newly diagnosed with GC (overall incidence rate: 7.6 ASR per 100,000), and 18,138 deaths were reported (mortality rate: 5.9 ASR per 100,000) due to GC in 2022 (1).

The etiology of GC and gastroesophageal junction cancer (GEJC) is heterogeneous and multifactorial, and major risk factors include genetic factors, *H. pylori* infection, diet, and lifestyle (2). *H. pylori* eradication, changes in lifestyle, and early detection, complemented with treatment, are the primary strategies for the prevention and management of GC and GEJC (2). Endoscopic ultrasonography computed tomography of the chest and abdomen, and biopsy are the routine diagnostic procedures for these conditions (3). Still, the asymptomatic nature of GC and GEJC during the early stages often delays diagnosis to an advanced stage in most patients (3).

Surgical resection remains the only curative option for GC and GEJC, often complemented by adjuvant or neoadjuvant chemotherapy and radiotherapy (4). However, the advancements in targeted therapies and immunotherapies have significantly expanded the treatment landscape (5). Currently, several therapies are approved for GC and GEJC, including trastuzumab (a first-line treatment combined with cisplatin-based chemotherapy for HER-2 positive tumors), pembrolizumab (used as first-line therapy for patients with unresectable or metastatic HER2-positive tumors and PD-L1 expression, in combination with trastuzumab, fluoropyrimidine, and platinum-based chemotherapy), and ramucirumab (a second-line therapy administered solo or alongside chemotherapy) (4, 6).

Emerging evidence highlights biomarkers such as PD-L1, MSI, HER-2, tumor mutation burden (TMB), and Epstein-Barr virus (EBV) as vital tools for detecting early tumors, evaluating prognosis, monitoring tumor burden, predicting drug resistance, and tailoring therapy decisions (7). These biomarkers increasingly play a pivotal role in identifying patient populations most likely to benefit from immunotherapy and targeted treatment approaches (8). Expanding research on biomarker distribution in GC and GEJC patients is essential for enabling clinicians to make more informed treatment decisions.

PD-L1 is a clinically important biomarker that can predict the response to immunotherapies and targeted therapies in patients with GC and GEJC (9). This transmembrane protein suppresses immune responses by inhibiting T-cell activation and cytokine secretion, reducing the proliferation of PD-1-positive malignant cells, and inducing apoptosis (10). The combined positive score (CPS), calculated as the total number of positive immune and tumor cells divided by the total viable tumor cells, multiplied by 100, is an effective method for evaluating PD-L1 expression. CPS scoring is particularly valuable in predicting responses to immunotherapy regimens such as pembrolizumab (11). Although there is no consensus (12, 13), research suggests CPS PD-L1 as an independent prognostic biomarker in patients with GC and GEJC (12, 14–16). A cohort study of Caucasian patients linked high PD-L1/PD-1 expression to significantly better outcomes, establishing PD-L1 as an independent prognostic factor for survival (17). Another study reported that the positive PD-L1 expression patients tend to have lower overall survival than the negative PD-L1 expression patients (18). Therefore, CPS PD-L1 plays a crucial role in guiding physicians in selecting patients for immunotherapy treatments.

Similar to PD-L1, MSI is a potential prognostic factor that can predict the survival of patients with GC or GEJC (9). MSI is caused by a defective DNA mismatch repair system, and has been observed in several cancer types, including GC and GEJC (19). Clinical trials have demonstrated that patients with MSI-high GC or GEJC could respond well to immunotherapy (20).

HER-2 is another potential prognostic biomarker though its prognostic value in GC and GEJC remains debated. Some studies concluded that HER-2-positivity may not be an independent prognostic factor for GC and GEJC (21, 22), while others associate HER-2-positivity with poorer survival (23, 24). Research shows approximately 85% of HER-2-positive GC cases are also PD-L1-positive when assessed using the PD-L1 antibody 22C3. The combined detection of the HER2 gene and PD-L1 in GC provides valuable insights for utilizing combination targeted therapies (25). Regardless of the role of HER-2 in the tumorigenesis, HER-2 expression predicts a better response to anti-HER-2-based therapies (26).

Although the importance of biomarker analysis for selecting a suitable immunotherapy or targeted therapy regimen is well established in patients with GC and GEJC, there are limited real-world data, especially in the Brazilian context. Comprehensive investigation into these biomarkers and their relationships with clinicopathological and demographic parameters is essential to address this research gap. Therefore, the present study aims to analyze PD-L1 expression among Brazilian patients with GC and GEJC and understand the relationship between PD-L1 expression and other biomarkers and potential confounders.

## 2 Methods

### 2.1 Study design and settings

This observational retrospective study examined PD-L1 expression, MSI and HER-2 status in patients with GC or GEJC at the *Barretos Cancer Hospital*, an oncological Hospital in Brazil (also known as *Hospital de Amor de Barretos*) between March 2019 and May 2019. This study was approved by the research ethics committee of *Barretos Cancer Hospital* (certificate of presentation number: 98723618.3.0000.5437) on 10 April 2018.

### 2.2 Patient selection

This study included adult patients (aged ≥18 years at diagnosis) diagnosed with GC or GEJC, confirmed either histologically or cytologically, and who had available medical records at the institution. Additionally, patients needed to have a formalin-fixed, paraffin-embedded (FFPE) GC or GEJC tissue sample collected at the time of diagnosis/surgery or relapse. Patients were excluded if they had another primary tumor after collecting the tumor sample for PD-L1 expression, MSI and HER-2 status evaluation or if their FFPE samples were collected over four years before the initiation of the study.

### 2.3 Data sources

Information on demography, pathology, treatments, other biomarkers, and clinical results were collected from the institution’s medical record system. PD-L1 expression in tumor samples was measured by the immune histochemical (IHC) method. If information on MSI and HER-2 status were not available for some tissue samples in the institution’s database, molecular assays were performed to determine their MSI status, and IHC and fluorescence *in situ* hybridization (FISH) assays were conducted to evaluate HER-2 expression.

### 2.4 Study outcomes

The primary outcomes of interest were PD-L1 expression levels and the potential relationships between PD-L1 expression and other biomarkers, such as MSI-high and HER-2 positivity.

The secondary study outcomes included patient demographics (age, sex, ethnicity, comorbidity), disease pathology (diagnosis date, type of cell histology, stage, grade, metastatic sites), treatment before relapse (drug regimen, start and end dates, reason for treatment discontinuation), and other biomarkers (*H. pylori*).

### 2.5 Primary Objectives - Evaluation of biomarkers of interest

#### 2.5.1 PD-L1 IHC assay

PD-L1 expression status was assessed by IHC assay using the PD-L1 IHC 22C3 PharmDx FDA-approved kit (Agilent Dako, Santa Clara, USA) and CPS as standard. All slides were stained on an automated IHC platform, Dako Automated Link 48, with an anti-PD-L1 antibody, clone 22C3 (Agilent Dako, Santa Clara, USA). Two trained pathologists interpreted the assay results. The staining criteria employed are described by the Interpretation Manual—Gastric or Gastro-Esophageal Junction Adenocarcinoma, Agilent Dako (Santa Clara, USA), 2019 (27). A threshold criterion for positive cases was established when CPS was equal to or higher than 1 and negative for cases lower than 1.

#### 2.5.2 MSI assay

DNA from FFPE tissues was retrieved from 10-µm slides after careful microdissection of the tumor area, ensuring more than 60% of neoplastic cells, as previously reported (28). DNA was isolated using the QIAamp DNA Mini Kit (Qiagen, Venlo, The Netherlands) following the manufacturer’s instructions, quantified by NanoDropVR 2000 (Thermo Scientific, Waltham), and stored at -20°C for further applications (28). MSI evaluation was performed using a multiplex PCR comprising six quasi-monomorphic and mononucleotide repeat markers (BAT25, BAT26, NR21, NR24, NR27, and HSP110) as reported (29). Cases with two or more markers out of the quasi monomorphic variation range (QMVR) were classified as MSI-high, cases with one marker out of the QMVR, were classified as MSI-low, and cases without markers out of QMVR were classified as MSS. MSI-high cases were considered MSI-positive, and MSI-low or MSS cases were classified as non-MSI-high, as reported by our group (29) and in line with current clinical research trends (30).

#### 2.5.3 HER-2 IHC assay

The IHC assay of HER-2 protein was performed on an automated IHC platform Benchmark Ultra—Ventana Roche (Oro Valley, USA) using the 4B5 antibody, Roche Tissue Diagnostics (Oro Valley, USA), and the UltraView Universal DAB Detection Kit. The antigens were retrieved inside the automated platform with the proprietary retrieval solution for 32 minutes at 95°C. The 4B5 rabbit monoclonal primary antibody was incubated for 12 minutes. After the completion of all reactions, all slides were counterstained according to the manufacturer’s protocol. HER2 reactions were evaluated under optical microscopy using scores 0, 1, 2, and 3 defined by Hoffmann et al. (31). All cases with a score of 2 were further tested using the FISH assay to confirm HER2 status.

#### 2.5.4 HER-2 FISH assay

The tissue was processed using the HER-2 FISH assay kit (ZytoLight SPEC ERBB2/CEN 17 Dual Color Probe, ZytoVision, Bremerhaven, Germany) according to the manufacturer’s protocol. Average HER-2 gene copy numbers and average chromosome 17 centromeres were evaluated by counting the number of signals in at least 20 interphase, non-overlapping carcinoma cell nuclei, and the HER2 gene was considered amplified if the HER-2-to-chomosome 17 centromere ratio was greater than 2.0 and not amplified when the ratio was <2.0, according to Sauter et al. (32).

### 2.6 Secondary objectives - demographic and clinicopathological characteristics

Demographic and clinicopathological data were extracted from the institution’s medical records using a standardized case report form. The investigator verified the quality and accuracy of the data. Continuous variables were summarized using mean, standard deviation (SD), minimum, and maximum values, while categorical variables were reported as absolute numbers and percentages.

### 2.7 Statistical analysis

Statistical analyses were performed using the SPSS software v.21.0 (SPSS, Chicago, IL). For continuous variables, descriptive statistics were reported as mean, standard deviation (SD), minimum and maximum values. For categorical variables, absolute numbers and percentages were computed. The association between CPS prevalence and PD-L1, HER-2, and MSI status was compared in subgroups based on clinicopathological features using the chi-square or Fisher’s exact test. For all statistical comparisons, a significance level of 5% was considered (p<0.05).

## 3 Results

### 3.1 Demographic and clinicopathological characteristics

A total of 162 patients with a diagnosis of GC or GEJC were included in this study whose FFPE tissues were available from the Pathology Department of *Barretos Cancer Hospital*. The demographic and clinicopathological features of the patients are presented in **Table 1**. The average (SD) age of the patients was 61 (11.3) years. Most of them were male (65.4%), of Caucasian origin (67.1%), and had a family history of cancer (62.6%). The primary site of tumor in the majority of patients was the stomach (92%), being the antrum region the most affected (42%), followed by the body (11.72%), cardia (7.4%), stump (6.80%), fundus (1.23%), and pylorus (0.63%), 8% were in other locations and 22.22% had missing locations. The GEJC was the primary site of tumor for 8% of the patients. Tumors were poorly differentiated in most patients (60.5%). According to tumor node metastasis (TNM, 8^th^ edition) staging, 44.4% of the patients had advanced stage III/IV tumors, and in 90.1% of the patients, the tumor had metastasized. In most of the patients, the *H. pylori* infection status was negative (86.0%).

**Table 1:** Clinicopathological features of patients with gastric and gastroesophageal junction cancer.

Variables	Category	Total = 162	
		n	(%)
Age (years)	Mean (SD)	61 (11.3)	
	Min–Max	25–87	-
Gender	Female	56	(34.6)
	Male	106	(65.4)
Ethnicity	Not White	53	(32.9)
	White	108	(67.1)
	Missing	1	-
Family history of cancer	No	58	(37.4)
	Yes	97	(62.6)
	Missing	7	–
Tumor site	Stomach	149	(92.0)
	Gastroesophageal junction	13	(8.0)
Tumor differentiation	Well differentiated	4	(2.50)
	Moderate differentiated	58	(35.8)
	Poorly differentiated	98	(60.5)
	Missing	2	(1.20)
TNM (8th Edition) staging	I/II	80	(55.6)
	III/IV	64	(44.4)
	Missing	18	-
Metastasis	Yes	146	(90.1)
	No	16	(9.90)
*Helicobacter pylori*	Negative	123	(86.0)
	Positive	20	(14.0)
	Missing	19	-

^SD, Standard deviation; TNM, Tumor node metastasis.^

### 3.2 PD-L1, MSI expression, and HER-2 status

**Figure 1:**
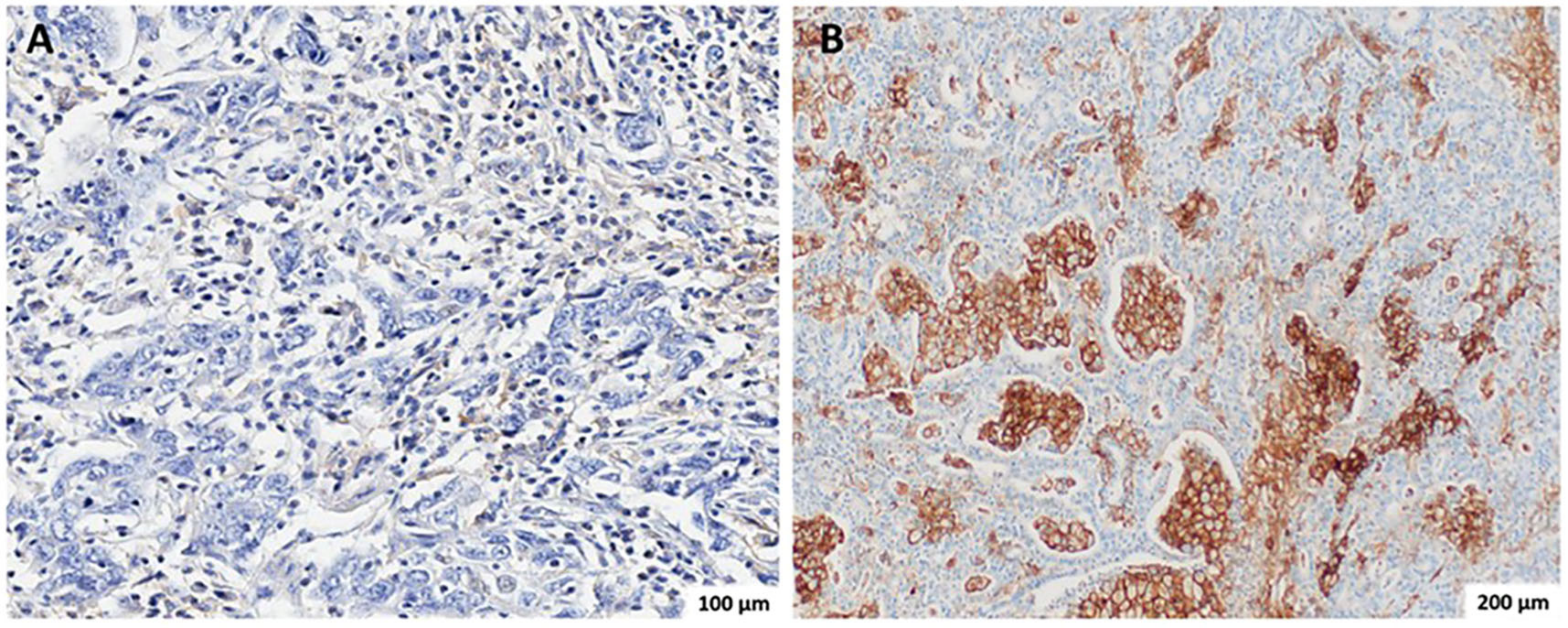
Representative immunohistochemistry (IHC) staining for PD-L1 in gastric adenocarcinoma using the PD-L1 IHC 22C3 pharmDx kit (Agilent Dako). **(A)** PD-L1–negative case (CPS < 1), showing absent or minimal brown membranous staining in tumor and immune cells. Image captured at 20× magnification; scale bar = 100 µm. **(B)** PD-L1–positive case with high CPS (≥50), showing intense and diffuse brown membranous staining in tumor and immune cells. Image captured at 10× magnification; scale bar = 200 µm. PD-L1 expression was assessed using the Combined Positive Score (CPS), calculated as the number of PD-L1–stained tumor and immune cells divided by the total number of viable tumor cells, multiplied by 100. CPS ≥ 1 was considered positive.

**Figure 2:**
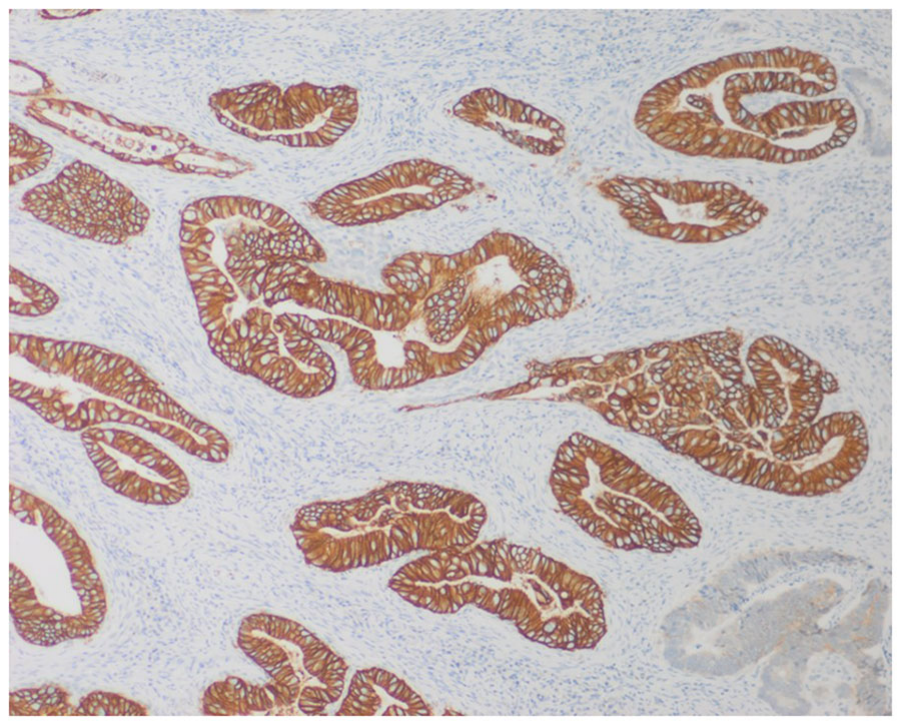
Representative immunohistochemistry (IHC) staining for HER-2 in gastric adenocarcinoma tissue using the monoclonal 4B5 antibody (Ventana, Roche). The image shows a case with a HER-2 IHC score of 3+, defined by intense, complete, and circumferential brown membranous staining in more than 10% of tumor cell clusters, indicating HER-2 overexpression. Image captured at 10× magnification; scale bar = 200 µm.

**Figure 3:**
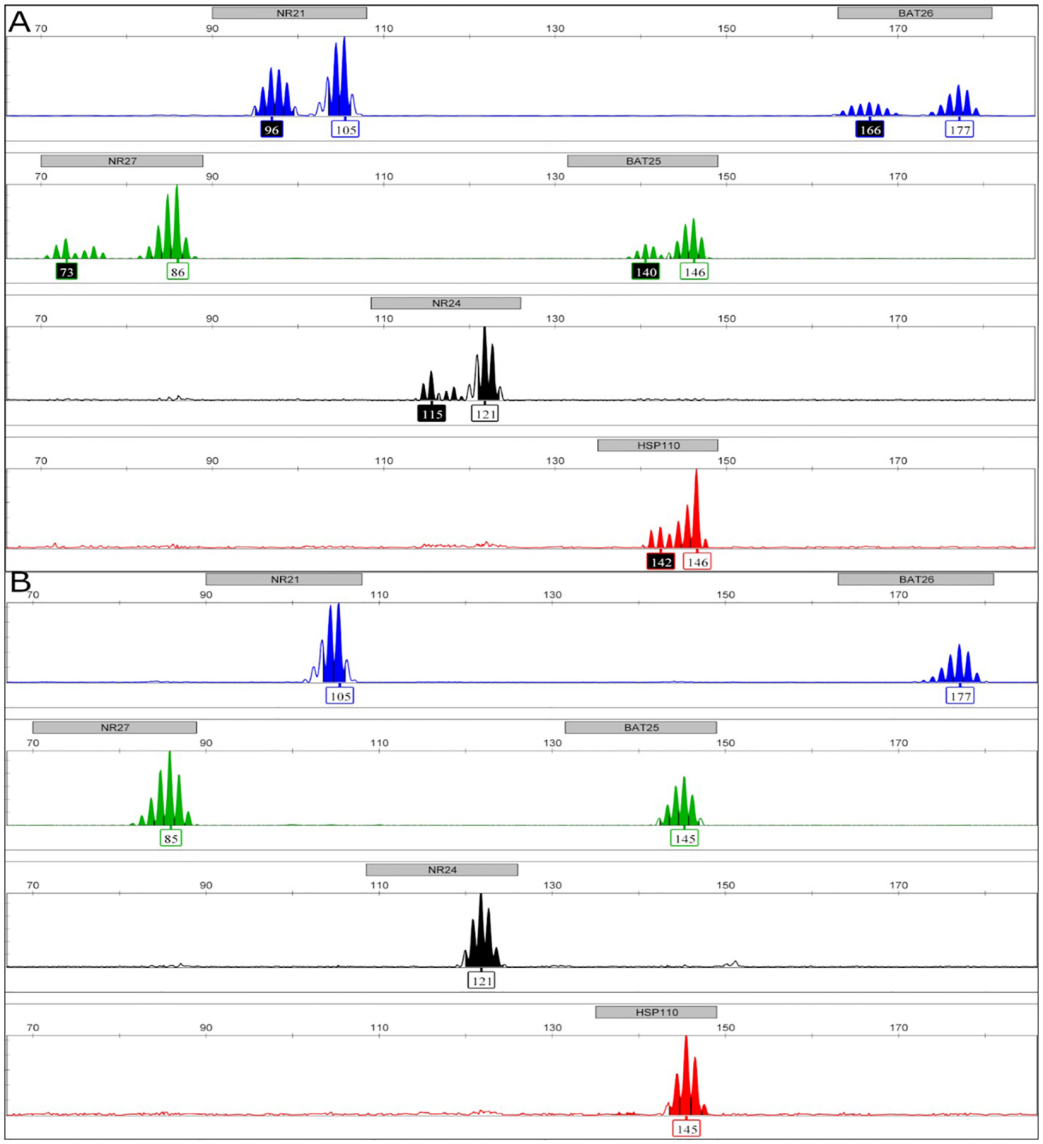
Representative figure of microsatellite instability (MSI) fragment analyses. Images obtained by GeneMapper Software version 4 (Thermo Scientific, Waltham) show each mononucleotide microsatellite marker (BAT25, BAT26, NR21, NR24, NR27, and HSP110) analyzed. The X-axis characterizes the fragment size in base pairs, and the Y-axis represents the fragment quantity in RFU (relative fluorescence unit). The considered normal length of each one is presented in the white rectangles. The numbers in the black rectangles represent fragments detected with altered lengths (unstable markers). **Figure 2A** exemplifies an MSI-High case, and **Figure 2B** exemplifies an MSS case.

**Table 2** summarizes the PD-L1, HER-2 expression, and MSI status of the FFPE tissue samples of the patients with GC or GEJC, as well as the association between these parameters and demographic and clinicopathologic characteristics. The IHC analysis for PD-L1 protein showed that 49.4% (80/162) had PD-L1-positive status (CPS ≥1) (**Table 2**). **Figure 1** shows representative images of tissue samples for the PD-L1 IHC analysis (**Figure 1A**: negative cases, **Figure 1B**: positive cases with CPS ≥50). MSI assay results showed the presence of MSI positive in 12.3% (20/162) of the patients. **Figure 2** illustrates the representative diagram of MSI markers for MSI and MSS samples. HER-2 expression was observed in 8.0% (13/161) of the patients. **Figure 3** shows representative images of tissue samples for IHC analysis for HER-2 positive expression.

**Table 2:** Association between clinicopathological and molecular features of patients with gastric and gastroesophageal junction cancer (n = 162).

Variable	Category	PD-L1					MSI status					HER-2				
		Negative (n = 82)		Positive (CPS≥1) (n = 80)		p-value	non-MSI-high (n = 142)		MSI-high (n = 20)		p-value	Negative (n = 148)		Positive (n = 13)		p-value
		n	(%)	n	(%)		n	(%)	n	(%)		n	(%)	n	(%)	
Age (years)	Mean (SD)	58		65		<0.001*	60		66		0.035*	61 (11)		**63** (13)		0.786
Gender	Female	31	(37.8)	25	(31.3)	0.412	49	(34.5)	7	(35.0)	≥0.99	52	(35.1)	3	(23.1)	0.545
	Male	51	(62.2)	55	(68.8)		93	(65.5)	13	(65.0)		96	(64.9)	10	(76.9)	
Ethnicity	Not White	23	(28.0)	30	(38.0)	0.240	45	(31.9)	8	(40.0)	0.612	51	(34.5)	2	(16.7)	0.340
	White	59	(72.0)	49	(62.0)		96	(68.1)	12	(60.0)		97	(65.5)	10	(83.3)	
Family history of cancer	No	29	(38.2)	28	(37.8)	≥0.99	49	(37.7)	8	(40.0)	≥0.99	50	(36.5)	6	(50.0)	0.368
	Yes	47	(61.8)	46	(62.2)		81	(62.3)	12	(60.0)		87	(63.5)	6	(50.0)	
Tumor site	Stomach	76	(92.7)	73	(91.3)	0.780	129	(90.8)	20	(100.0)	0.372	137	(92.6)	11	(84.6)	0.282
	Gastro-esophageal junction	6	(7.3)	7	(8.8)		13	(9.2)	0	(0.0)		11	(7.4)	2	(15.4)	
Tumor differentiation	Well differentiated	1	(1.3)	3	(3.8)	0.057*	4	(2.9)	0	(0.0)	0.334	4	(2.7)	0	(0.0)	0.052
	Moderate differentiated	23	(28.8)	35	(43.8)		53	(37.9)	5	(25.0)		48	(32.9)	9	(69.2)	
	Poorly differentiated	56	(70.0)	42	(52.5)		83	(59.3)	15	(75.0)		94	(64.4)	4	(30.8)	
TNM staging	I/II	39	(52.7)	41	(58.6)	0.506	68	(54.4)	12	(63.2)	0.622	76	(57.1)	4	(36.4)	0.217
	III/IV	35	(47.3)	29	(41.4)		57	(45.6)	7	(36.8)		57	(42.9)	7	(63.6)	
Metastasis	No	70	(85.4)	76	(95.0)	0.063	129	(90.8)	17	(85.0)	0.422	133	(89.9)	12	(92.3)	≥0.99
	Yes	12	(14.6)	4	(5.0)		13	(9.2)	3	(15.0)		15	(10.1)	1	(7.7)	
Helicobacter Pylori	Negative	63	(51.2)	9	(45.0)	0.638	107	(87.0)	18	(90.0)	≥0.99	113	(86.3)	9	(81.8)	0.654
	Positive	60	(48.8)	11	(55.0)		16	(13.0)	2	(10.0)		18	(13.7)	2	(18.2)	
PD-L1	Negative	NA	NA	NA	NA	NA	79	(55.6)	3	(15.0)	<0.001*	77	(52.0)	5	(38.5)	0.397
	Positive	NA	NA	NA	NA		63	(44.4)	17	(85.0)		71	(48.0)	8	(61.5)	
MSI status	MSS	79	(96.3)	63	(78.8)	0.001**	NA	NA	NA	NA	NA	128	(86.5)	13	(100)	0.373
	MSI	3	(3.7)	17	(21.3)		NA	NA	NA	NA		20	(13.5)	0	(0.0)	
HER-2	Negative	77	(93.9)	71	(89.9)	0.397	128	(90.8)	20	(100)	0.225	NA	NA	NA	NA	NA
	Positive	5	(6.1)	8	(10.1)		13	(9.2)	0	(0.0)		NA	NA	NA	NA	

*Chi-square association test; **Fisher exact test.

HER-2, Human epidermal growth factor receptor 2; MSI, Microsatellite instability; NA, Not applicable. PD-L1, Programmed cell death ligand 1.

SD, Standard deviation; TNM, Tumor node metastasis.

Bold: Statistically significant.

### 3.3 Association between PD-L1, MSI status, and HER-2 expression and demographic and clinicopathologic characteristics

The association analysis revealed that PD-L1 expression was significantly associated with older age (p<0.001) and MSI-high status (p<0.001), whereas a tendency for association was observed with tumor differentiation status (p = 0.057) and metastasis status (p = 0.063) (**Table 1**). Similar to PD-L1 expression, MSI-high status was significantly associated with older age (p = 0.035). However, no statistically significant association was found between any other clinicopathological characteristics and PD-L1 expression, HER-2 expression, or MSI-high status (**Table 2**).

## 4 Discussion

The present investigation is one of the very few studies conducted among Brazilian patients with GC and GEJC, adding valuable evidence on the prevalence of three critical biomarkers: CPS PD-L1, MSI, and HER-2. It further explores their associations with clinicopathological features, contributing to the understanding of prognosis and therapeutic response.

Previously studies have investigated PD-L1 expression in patients with GC and GEJC (12, 33–36). However, a 2019 review summarizing PD-L1 expression among patients with GC indicated significant variability in this protein’s expression, ranging from 9% to 72% across different studies (36) that could be due to methodological differences, such as antibody clones, cutoff values, and evaluation methods. Even studies that used the CPS method for PD-L1-positivity scoring showed a great variation (15.8%–80% of patients with GC were detected to have PD-L1 expression), possibly due to the differences in the antibody used for PD-L1 staining (22C3 antibody or 28–8 antibody) (12, 33–35). In studies using CPS with the 22C3 antibody, 49%–57% of GC patients were CPS-positive (37–39), consistent with our finding of 49.4% CPS PD-L1-positivity.

In addition to the CPS ≥1 threshold used in this study, it is important to consider the implications of higher CPS thresholds (e.g., ≥10 and ≥50) in the context of immunotherapy responsiveness. Several clinical trials and regulatory decisions have demonstrated that patients with higher CPS scores may derive greater benefit from immune checkpoint inhibitors, particularly pembrolizumab (40). For instance, the KEYNOTE-059 and KEYNOTE-061 trials highlighted improved outcomes in patients with CPS ≥10, supporting the clinical relevance of stratifying PD-L1 expression beyond the CPS ≥1 cutoff 1. Although our dataset did not stratify PD-L1 expression beyond the CPS ≥1 threshold, future analyses incorporating these higher thresholds could provide more nuanced insights into patient selection and therapeutic outcomes. This stratification approach is especially relevant for tailoring therapies to Brazilian patients, whose biomarker prevalence may differ from global averages (38).

The MSI-high status in patients with GC, detected in 12.3% of patients in this study, is associated with better prognostic outcomes and higher overall survival rates, as supported by systematic review and meta-analyses (41) (42). Previous studies reported that the prevalence of MSI ranged from 8% to 33% (36, 43), with our study reporting 12.3%. Similar to the present study, a Brazilian study reported that 21% of GC and GEJC patients were MSI and that 54% of all patients had stage I or II disease (12). This variability can be attributed to variations in the proportions of patients at different stages of GC and different MSI assessment time, which is predominantly conducted at the time of surgery rather than at diagnosis according to the systematic review and meta-analysis conducted by Petrelli et al. (41). The strong association observed between MSI-high status and PD-L1 expression reinforces the value of dual biomarker testing in guiding immunotherapy decisions.

HER-2 positivity was observed in 8% of patients, which is lower than global averages but consistent with other Brazilian studies that have reported ranges from 6% to 16%, aligning with our study results (44–49). A systematic review reported that HER-2 positivity rates reported in articles from Asian (19.52%) countries were quantitatively higher than those from European (16.91%) areas, and the only Brazilian study included reported a 10.5% rate of HER-2 positivity (44). Another multinational study reported a similar rate of HER-2 positivity (22%) among metastatic GC (50). Overall, the prevalence of HER-2-positivity is lower among Brazilian patients compared with patients from other geographies, suggesting that fewer patients with GC from Brazil may be eligible for anti-HER-2 therapies. However, further studies are required to support this hypothesis, as the determination of HER-2-positivity is largely dependent on study settings.

Variability in HER-2 prevalence may be attributed to methodological differences in IHC protocols and scoring systems, as well as regional tumor biology influenced by genetic ancestry and environmental exposures (44). Methodologically, differences in IHC protocols, antibody clones, scoring systems, and interpretation criteria can significantly influence HER-2 detection rates. For instance, variability in fixation times, tissue processing, and the use of whole-tissue sections versus tissue microarrays may lead to under- or overestimation of HER-2 expression (44). Biologically, regional heterogeneity in tumor biology, including genetic ancestry and environmental exposures, may also contribute to the lower prevalence observed in Brazilian cohorts. Studies have suggested that HER-2 overexpression may be less frequent in populations with higher proportions of diffuse-type gastric cancer, which is more common in Latin America (46, 47). Standardizing HER-2 testing methodologies and considering regional biological factors are crucial for accurate prevalence assessment and treatment planning.

The study revealed a strong association between PD-L1 expression and MSI-high status, a finding that aligns with previous research (51). MSI tumors are hypermutated, and they produce neoantigens, which attract millions of T lymphocytes and augment the expression of PD-L1 through gamma interferon secretion (36). While such a correlation between PD-L1 expression and MSI-high status is absent in many other cancer types (52), it has been consistently reported in studies focusing on GC (12, 52, 53). Regarding treatment, pembrolizumab is US FDA-approved for advanced PD-L1 positive gastric adenocarcinoma (54). It has demonstrated a high tumor response rate among patients with positive MSI-high status (55). Furthermore, older age is a common factor identified in this study to be associated with PD-L1 expression and MSI-high status. This correlation is consistent with findings reported in studies conducted among Brazilian and global populations (38, 56, 57).

This study contributes novel insights to the Brazilian GC literature by expanding the understanding of biomarker prevalence and associations in a real-world setting. While prior studies have explored PD-L1 expression in resectable GC, our investigation uniquely evaluates PD-L1 using the CPS method with the 22C3 antibody in a broader cohort that includes both GC and GEJC across all disease stages. Furthermore, we concurrently assess MSI and HER-2 status and their associations with clinicopathological features, offering a more comprehensive biomarker landscape. Notably, the observed strong correlation between PD-L1 expression and MSI-high status reinforces emerging evidence of immunogenic tumor profiles in this population and supports the clinical relevance of dual biomarker testing to guide immunotherapy decisions in Brazilian patients.

### 4.1 Limitations

Although this study adds valuable information regarding the levels of important biomarkers and their correlation with clinicopathologic characteristics in Brazilian patients with GC and GEJC, these results should be interpreted in the context of its limitations. Although it is crucial to evaluate PD-L1 expression accurately by IHC in clinical practice, the frequency of PD-L1 expression and its association with prognosis can vary due to several factors such as the antibody clone, the preparation of tissue samples, the evaluation system, tumor heterogeneity, and geographical differences of the recruited patients (15, 58).

It is important to note that the study has some other limitations, including the small sample size and single-center settings. Sample size is a crucial factor in research as it directly impacts the reliability and extent to which the findings can be generalized to the larger population. While larger sample sizes yield smaller margins of error and are more representative, a sample size that is too large may significantly increase the cost and time taken to conduct the research. Increasing the sample size improves the likelihood of finding a statistically significant effect. In contrast, effect sizes are independent of the sample size. Due to these limitations, the results of this study can not be generalized to the entire Brazilian population of patients with GC and GEJC. In addition, the number of clinicopathological characteristics and biomarkers analyzed was limited; hence, there might have been important correlations that remain unidentified. Finally, this study may have other limitations inherent to all observational and retrospective studies with secondary data, including selection bias, unobserved confounding factors, missing data among others.

## 5 Conclusion

This study provides important real-world insights into the prevalence and clinical relevance of key biomarkers—PD-L1, MSI, and HER-2—in Brazilian patients with GC and GEJC. We observed a high rate of PD-L1 expression (CPS ≥1) and a strong association with MSI-high status, reinforcing the value of these biomarkers in guiding immunotherapy decisions. In contrast, HER-2 positivity was lower than global averages but consistent with other Brazilian studies, suggesting potential regional or methodological influences. These findings highlight the need for standardized testing protocols and underscore the importance of considering local tumor biology in treatment planning. Future research should focus on expanding cohort sizes, incorporating longitudinal data, and evaluating additional biomarkers such as tumor mutational burden and Epstein-Barr virus. Stratifying PD-L1 expression at higher CPS thresholds (e.g., ≥10, ≥50) may also refine patient selection for immunotherapy. These directions can support more precise, biomarker-driven treatment strategies and inform future research in diverse clinical settings.

## Data Availability

The raw data supporting the conclusions of this article will be made available by the authors, without undue reservation.
